# Prolonged extracorporeal membrane oxygenation therapy for severe acute respiratory distress syndrome in a child affected by rituximab-resistant autoimmune hemolytic anemia: a case report

**DOI:** 10.1186/1752-1947-3-6443

**Published:** 2009-04-01

**Authors:** Chiara Beretta, Veronica Leoni, Mario Renato Rossi, Momcilo Jankovic, Nicolo Patroniti, Giuseppe Foti, Ettore Biagi

**Affiliations:** 1 Department of Pediatric Hematology, San Gerardo Hospital, Monza, University of Milan-Bicocca, Milan, Italy; 2 Department of Intensive Care, San Gerardo Hospital, Monza, University of Milan-Bicocca, Milan, Italy

## Abstract

**Introduction:**

Autoimmune hemolytic anemia in children younger than 2 years of age is usually characterized by a severe course, with a mortality rate of approximately 10%. The prolonged immunosuppression following specific treatment may be associated with a high risk of developing severe infections. Recently, the use of monoclonal antibodies (rituximab) has allowed sustained remissions to be obtained in the majority of pediatric patients with refractory autoimmune hemolytic anemia.

**Case presentation:**

We describe the case of an 8-month-old Caucasian girl affected by a severe form of autoimmune hemolytic anemia, which required continuous steroid treatment for 16 months. Thereafter, she received 4 weekly doses of rituximab (375 mg/m^2^/dose) associated with steroid therapy, which was then tapered over the subsequent 2 weeks. One month after the last dose of rrituximab, she presented with recurrence of severe hemolysis and received two more doses of rrituximab. The patient remained in clinical remission for 7 months, before presenting with a further relapse. An alternative heavy immunosuppressive therapy was administered combining cyclophosphamide 10 mg/kg/day for 10 days with methylprednisolone 40 mg/kg/day for 5 days, which was then tapered down over 3 weeks. While still on steroid therapy, the patient developed an interstitial pneumonia with Acute Respiratory Distress Syndrome, which required immediate admission to the intensive care unit where extracorporeal membrane oxygenation therapy was administered continuously for 37 days. At 16-month follow-up, the patient is alive and in good clinical condition, with no organ dysfunction, free from any immunosuppressive treatment and with a normal Hb level.

**Conclusions:**

This case shows that aggressive combined immunosuppressive therapy may lead to a sustained complete remission in children with refractory autoimmune hemolytic anemia. However, the severe life-threatening complication presented by our patient indicates that strict clinical monitoring must be vigilantly performed, that antimicrobial prophylaxis should always be considered and that experienced medical and nursing staff must be available, to deliver highly specialized supportive salvage therapies, if necessary, during intensive care monitoring.

## Introduction

Autoimmune hemolytic anemia (AIHA) in children is usually characterized by a severe course with a mortality rate of approximately 10% [1]. The required prolonged immunosuppressive therapy often leads to steroid dependence [2]. The administration of non-steroidal immunosuppressive drugs such as cyclosporine A, cyclophosphamide and azathioprine, has been used in the past [1]-[4]. Nowadays, the use of monoclonal antibodies such as rituximab, has given promising results for pediatric refractory AIHA [5]-[7], with sustained remissions in the majority of patients. Nevertheless, potentially life-threatening infections are known to occur with rituximab [7]. In the event of rituximab failure, there is no general consensus or guidelines available indicating precisely how to manage resistant forms of AIHA. Heavy immunosuppression consisting of the combined use of cyclophosphamide and high-dose steroids may be considered [8,9].

## Case presentation

**Figure 1:**
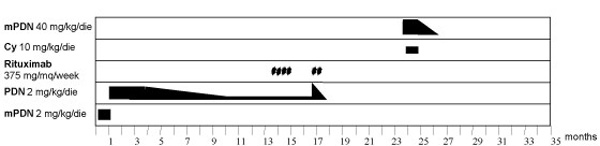
**Immunosuppressive therapy administered during the course of refractory autoimmune hemolytic anemia**. mPDN, methylprednisolone; Cy, cyclophosphamide; PDN, prednisone.

We report the case of an 8-month-old Caucasian girl referred to us for observation due to intense pallor, jaundice, lethargy and fever. Serological evaluations revealed severe anemia (Hb = 2.8 g/dL) with a strongly positive direct antiglobulin test and high-titer warm IgG autoantibody. AIHA was diagnosed and steroid therapy with intravenous methylprednisolone at 2 mg/kg/day was administered for 5 days (Figure 1). An adequate Hb increase was obtained and the child was discharged after 10 days with oral prednisone at 2 mg/kg/day.

During the subsequent months, several attempts were made to taper off the prednisone, but the patient had developed steroid dependence. Considering this dependence on high steroid doses, a therapeutic course with four doses of rituximab was performed (375 mg/m^2^/dose) at weekly intervals (Figure 1). Before rituximab infusion, serum immunoglobulin levels were normal and subpopulation lymphocyte counts were within the normal range. The treatment with rituximab was well tolerated and the patient received intravenous substitutive therapy with commercially available immunoglobulin preparations (400 mg/kg, every 3 weeks for 6 months). One month after the end of the first course of rituximab, while still receiving low-dose steroids, the patient presented with a clinical relapse of AIHA, so prednisone was increased to 2 mg/kg/day and two further rituximab infusions were performed (Figure 1). After these infusions, B lymphocytes became undetectable and the count returned to normal values 8 months after treatment. The patient remained in clinical remission and free from immunosuppressive drugs for 7 months, before presenting with a further relapse. A more intensive treatment was performed (Figure 1) with cyclophosphamide 10 mg/kg/day for 10 days and methylprednisolone 40 mg/kg/day for 5 days, which was tapered over 20 days. Hb level increased and the patient was discharged 10 days later in good clinical condition, without any antifungal or antiviral prophylaxis.

**Figure 2:**
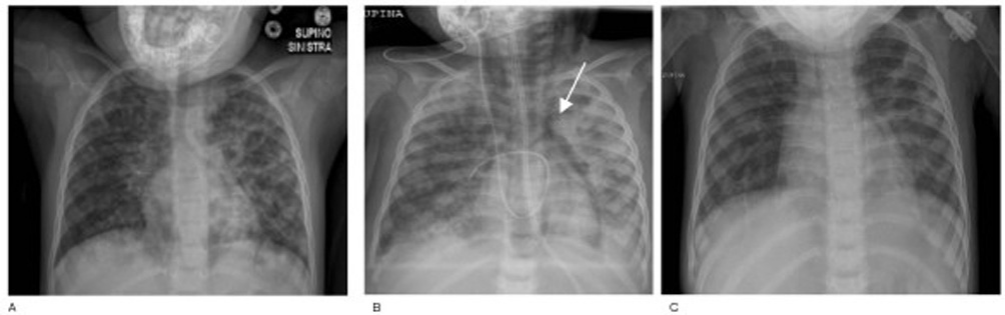
**Chest X-ray film sequence**. **(A)** Day of admission to the intensive care unit; **(B)** before extracorporeal membrane oxygenation with evidence of pneumomediastinum (white arrow), in spite of protective ventilatory strategy; **(C)** before intensive care unit discharge showing a complete resolution of acute respiratory distress syndrome.

**Figure 3:**
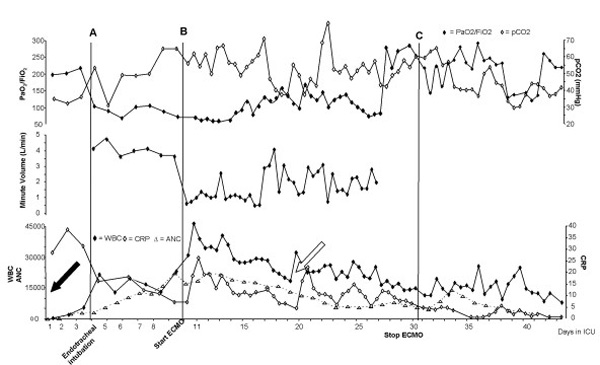
**Main gas exchange (upper panel: PaO_2_/FiO_2_, and PaCO_2_), ventilatory (middle panel: minute ventilation), and laboratory (lower panel: white blood cell count WBC, absolute neutrophil count ANC, and C reactive protein CRP) data in intensive care unit**. Vertical solid line refers to: **(A)** endotracheal intubation; **(B)** connection to extracorporeal membrane oxygenation; **(C)** disconnection from extracorporeal membrane oxygenation. The black arrow indicates the first positivity for *Aspergillus galactomannan* antigen in the patient's serum. The white arrow indicates the first microbiological evidence of *Pseudomonas aeruginosa*.

Two weeks later, the child was referred to the Emergency Room for respiratory failure, persistent fever and abdominal pain. Laboratory examination showed an Hb level of 12.8 g/dL, total leukocyte count (WBC) of 710/μL, absolute neutrophil count (ANC) of 90/μL, a platelet count (PLT) of 339,000/μL, and low levels of immunoglobulin (IgG = 360 mg/dL, IgA = 10 mg/dL, IgM = 33 mg/dL). Chest X-ray and CT scan revealed an interstitial pneumonia (Figure 2). Therapy with amikacin, ceftazidime, G-CSF and voriconazole was started. Within a few hours, her clinical condition deteriorated and the patient developed Acute Respiratory Distress Syndrome (ARDS), which required immediate admission to the intensive care unit (ICU). Acceptable gas exchange was initially maintained by non-invasive continuous positive airway pressure (Figure 3). Serologic tests showed a level of *Aspergillus galactomannan* antigen of 0.8. All tested virus and microbial antigens were negative. On day 4, concomitantly with an elevation of WBC from 400 to 10,400/μL (ANC = 4900/μL), respiratory conditions precipitated and endotracheal intubation and mechanical ventilation were started (Figure 3). A protective ventilatory strategy with tidal volume of 6 mL/kg and positive end expiratory pressure (PEEP) of 10 cmH_2_O was instituted.

In the following days, gas exchange deteriorated and PEEP levels rose to 17 cmH_2_O. Recruitment manoeuvres, prone positioning, and high doses of inhaled nitric oxide (NOi) were necessary to maintain viable gas exchange. Endotracheal instillation of porcine surfactant and a trial with High Frequency Oscillation were ineffective. On day 10, owing to the refractory hypoxia, worsening hypercapnia, and chest X-ray evidence of a pneumomediastinum, the patient was placed on venous-venous extracorporeal membrane oxygenation (ECMO) (Figure 3). A double lumen 15 french catheter (Maquet, Jostra Medizintechnik AG, Hirrlingen, Germany) was inserted into the right internal jugular vein. The ECMO circuit consisted of a polymethylpentene membrane oxygenator, permanent life support and a centrifugal Rotaflow pump (Maquet, Jostra Medizintechnik AG, Hirrlingen, Germany). ECMO was started with a blood flow of 0.8 to 0.9 L/minute and gas flow of 1 L/minute of 100% oxygen. Following the institution of ECMO, respiratory rate decreased from 45 to 10 breaths/minute, and it was possible to stop NOi.

After commencing caspofungin with voriconazole, WBC, ANC and C reactive protein (CRP) slowly decreased, while pulmonary function slightly improved. On day 19, a multidrug-resistant Pseudomonas aeruginosa was isolated from bronchoaspirate (Figure 3). In spite of antibiotic reinforcement with levofloxacin, Pseudomonas aeruginosa antibiogram showed increased resistance to all antibiotics and to colimicine which was started on day 26. On day 28, a sudden increase in resistance on the return part of the circuit caused a massive thrombosis in the oxygenator. The entire circuit and the cannula were immediately changed and ECMO restarted within 2 hours. Following the development of pulmonary embolism, the gas exchange rapidly worsened and NOi had to be restarted. An echocardiographic assessment showed right ventricular dilatation, with paradoxical septal wall motion and pulmonary hypertension (systolic pressure 90 mmHg). Prostacyclin and sildenafil improved the right heart function and effectively attenuated pulmonary hypertension. In the following days, WBC, ANC and CRP slowly decreased, while pulmonary function improved. Thirty days from ICU admission, ECMO was stopped, the patient rapidly restored her spontaneous ventilatory functions and she was extubated 10 days later. She was finally discharged from the ICU on day 44. At 16-month follow-up, the patient is alive and free from immunosuppressive drugs. At the time of last follow-up, Hb level was 13.5 g/dL, reticulocyte count was 11 × 10^9^/L, with total bilirubin, lactic dehydrogenase and haptoglobin all within the normal ranges.

## Discussion

AIHA in children younger than 2 years is in some cases characterized by a resistance to corticosteroids or dependence on high steroid doses and subsequent development of severe side effects [2]. Splenectomy or immunomodulating agents have frequently been used, but there is no consistent demonstration of their efficacy in controlling hemolysis [3]. Immunosuppressive drugs such as azathioprine, cyclosporine A, or cyclophosphamide, alone or in combination reduce steroid dependence and sometimes control hemolysis [1]-[4]. Clinical experience with monoclonal antibodies appears encouraging. In particular, rituximab is increasingly being used off-label, for difficult-to-treat auto-immune diseases and presents the advantage of inducing a selective B-cell depletion, sparing cellular immunity mediated by T cells and natural killer cells. Even though prospective controlled studies are not currently available, the efficacy of rituximab has been shown in pediatric studies. Quartier* et al.*[5] treated five pediatric refractory AIHA patients, who achieved a complete remission within 15 to 22 months after rituximab therapy. These results were confirmed by Zecca* et al.*[6] in a group of 15 children treated with rituximab. Four other children were treated by Motto *et al.*[7], with the achievement of complete remission. Nevertheless, the prolonged impairment of antibody production leads to an increased risk of viral and bacterial infections. For this reason, monthly intravenous immunoglobulin infusions are recommended for a minimum of 6 months following completion of therapy and prophylaxis for *P. jirovecii* pneumonia is also suggested [7].

The patient described in our report received four rituximab infusions in an off-label setting, followed by two additional doses over 6 months. Clinical remission was achieved for 7 months after which it was possible to interrupt steroid treatment. The pattern of immune reconstitution after rituximab therapy revealed persistently low immunoglobulin levels, partially corrected by the substitutive therapy. Immunoglobulin levels reached their normal range in 18 months and the lymphocyte subpopulations returned to normal range in 16 months. It is, nevertheless, difficult to quantify the real role of rituximab in this heavy immunosuppression, since a combined therapy with high doses of methylprednisolone and cyclophosphamide was subsequently started. Even though our patient did not present any early side effects related to the rituximab infusions, a prolonged follow-up should be carried out to monitor and prevent long-term side effects of rituximab, which are still unknown.

When our patient relapsed, an alternative treatment was required, since therapies with steroids, rituximab and intravenous immunoglobulins proved to be ineffective. The role of splenectomy in refractory AIHA is still controversial [1]-[4]. Although effective vaccinations are available, this surgical treatment should be avoided in children younger than 6 years of age, due to the risk of developing severe bacterial infections. According to local policy, drug-based immunosuppressive therapy is to be preferred. We therefore decided to adopt a combined therapy approach, with high doses of methylprednisolone (40 mg/kg/day for 5 consecutive days), which was then tapered down over 20 days, and cyclophosphamide (10 mg/kg/day for 10 consecutive days). This approach appeared to be feasible and encouraging, since we had previously successfully treated two pediatric cases of refractory AIHA with an identical approach [8,9]. The administration of methylprednisolone and cyclophosphamide increased the already significant immunodepression which had resulted from prior therapies and further contributed to the severity of the infectious complication presented by our patient that required ECMO therapy. While in the ICU, the patient underwent various ventilatory treatments, some of which are not considered conventional. Modern ventilatory strategy in ARDS aims to provide viable gas exchange with high oxygen concentration and PEEP, while minimizing the injurious effects of mechanical ventilation by using low tidal volume ventilation (6 mL/kg) [10]. Although other techniques such as the prone position, NOi and recruitment manoeuvres are effective in improving gas exchange, they did not prove effective in terms of survival [10]. Nevertheless, before ECMO, the only means of providing minimal acceptable oxygenation was to use both NOi and the prone position. Despite using low tidal volumes, a respiratory rate of up to 45 breaths/minute was necessary to obtain acceptable CO_2_ levels, and the occurrence of pneumomediastinum demonstrated that we were unable to provide an effective protective ventilatory strategy. Thus, ECMO was the only real means of providing such a strategy, while allowing adequate gas exchange.

Refractory AIHA in pediatric patients is a challenging disease that forces us to weigh up the risks and benefits of heavy and prolonged immunosuppressive therapies that can reduce or even eradicate the hemolysis, despite the risk of infectious complications. For this reason, we feel that prolonged viral and fungal prophylaxis therapy should always be considered, during and after the immunosuppressive therapy. Furthermore, strict clinical monitoring should be carried out, even when no evident symptoms are present. In our patient, we did not administer any prophylaxis and clinical monitoring was probably delayed for too long after discharge. Resolution of the infectious complication was possible thanks to an advanced intensive care assistance, which consisted of ECMO and the management of its related complications.

## Conclusions

This case study shows that rituximab-resistant AIHA in young children represents a significant challenge, requiring aggressive immunosuppressive therapy, which may potentially cause severe life-threatening complications. Nowadays, it is not clear which is the best immunosuppressive agent to be administered in the event of rituximab failure. We found that the combination of methylprednisolone and cyclophosphamide could be a valid alternative, based on previous experience. Nevertheless, a universal therapeutic flow-chart is still lacking and should be defined, which considers new therapeutic strategies such as alemtuzumab [11] or hematopoietic stem cell transplantation [12]. What is clear, however, in the case of heavy immunosuppressive therapy, is the importance of strict patient monitoring during and after immunosuppressive therapy and an antimicrobial prophylaxis, particularly for fungal agents and *P. jirovecii*.

## Abbreviations

AIHA: autoimmune hemolytic anemia; ANC: absolute neutrophil count; CT: computed tomography; WBC: leukocyte count; PLT: platelet count; ARDS: acute respiratory distress syndrome; ICU: intensive care unit; PEEP: positive end exxpiratory pressure; NOi: inhaled nitric oxide; ECMO: extracorporeal membrane oxygenation; CRP: C reactive protein.

## Consent

Written informed consent was obtained from the patient's parents for publication of this case report and any accompanying images. A copy of the written consent is available for review by the Editor-in-Chief of this journal.

## Competing interests

The authors declare that they have no competing interests.

## Authors' contributions

CB was the major contributor in collecting the patient's data and writing the manuscript. She gave final approval of the version to be published. VL made a substantial contribution in the data-analysis and interpretation, and has been involved in drafting the manuscript. She gave final approval of the version to be published. MRR was the major contributor in conception of the manuscript; he also revised the manuscript critically for important intellectual content. He gave final approval of the version to be published. MJ made a substantial contribution in the manuscript drafting and in revising it critically for important intellectual content. He gave final approval of the version to be published. NP was a contributor in acquisition of patient's data during the ICU admission and has been involved in drafting the manuscript for the part concerning the ICU admission. He gave final approval of the version to be published. GF made a substantial contribution in analysing and interpreting the patient's data during the ICU admission and has been involved in drafting the manuscript for the part concerning the ICU admission. He gave final approval of the version to be published. EB took direct medical care of the patient and was the major contributor in revising the manuscript critically for important intellectual content. He gave final approval of the version to be published
